# Quality of life and life satisfaction in long-term survivors of acute myeloid leukemia

**DOI:** 10.1038/s41375-025-02735-y

**Published:** 2025-08-22

**Authors:** Eva Telzerow, Dennis Görlich, Cristina Sauerland, Maja Rothenberg-Thurley, Anna Sophia Moret, Simon M. Krauß, Friederike H. A. Mumm, Susanne Amler, Wolfgang E. Berdel, Bernhard J. Wörmann, Utz Krug, Jan Braess, Pia Heußner, Wolfgang Hiddemann, Karsten Spiekermann, Klaus H. Metzeler

**Affiliations:** 1 Department of Medicine III and Comprehensive Cancer Center (CCC Munich LMU), LMU University Hospital Munich, Munich, Germany; 2 Institute of Biostatistics and Clinical Research, University of Muenster, Muenster, Germany; 3 Laboratory for Leukemia Diagnostics, Department of Hematology and Oncology, University Hospital, LMU Munich, Munich, Germany; 4 Department of Hematology, Cell Therapy, Hemostaseology and Infectious Diseases, Comprehensive Cancer Center Central Germany (CCCG), University of Leipzig, Leipzig, Germany; 5 Friedrich-Loeffler-Institute, Greifswald-Insel Riems, Germany; 6 Department of Medicine A, Hematology, Oncology and Pneumology, University Hospital Muenster, Muenster, Germany; 7 Department of Hematology, Oncology and Tumor Immunology, Charité University Medicine, Berlin, Germany; 8 DKMS Collection Center, Cologne, Germany; 9 Department of Oncology and Hematology, Hospital Barmherzige Brüder, Regensburg, Germany; 10 Department of Internal Medicine/Psycho-Oncology, Hospital Garmisch-Partenkirchen, Garmisch-Partenkirchen, Germany

**Keywords:** Acute myeloid leukaemia, Quality of life, Acute myeloid leukaemia

## Abstract

We performed a questionnaire-based cross-sectional study to analyze Acute Myeloid Leukemia (AML) long-term survivor (LTS) outcomes, including psychosocial well-being and somatic health status. Four-hundred-twenty-seven former AML patients participated (response rate, 63%) ≥5 years[y] and up to 18.6 y past their leukemia diagnosis (median, 11.3 y). Median age at study participation was 61 y (range 28y–93y), 23% had experienced disease relapse, and 63% had received allogeneic hematopoietic stem cell transplantation (alloHSCT). Overall, quality of life (QoL) and general life satisfaction (gLS) summary scores were higher in AML LTS (*p* < 0.001) compared to age-/sex-matched reference cohorts, although differences were small and likely not clinically relevant. However, we identified subgroups of survivors reporting impaired QoL (27%), gLS (13%) and health-related life satisfaction (hrLS; 17%). Using multivariable regression models, we identified predisposing and protective factors for each of these outcomes. Treatment with alloHSCT did not adversely impact QoL, gLS, or hrLS. In summary, global QoL and LS in AML LTS are comparable to the general population, irrespective of treatment modality, although some survivors report clinically significant impairment of global QoL and/or in specific domains. We identified factors associated with impaired outcomes (e.g., comorbidity and fatigue), delineating a subgroup of survivors with unmet needs ≥5 y after their AML diagnosis.

## Introduction

The proportion of patients with acute myeloid leukemia (AML) becoming long-term survivors (LTS), commonly defined as those surviving for 5 years or longer past their initial diagnosis [1], has increased over the last four decades [2–5]. Approximately half of patients <60 years, and a smaller proportion of older patients, can now expect to become LTS. As disease outcomes are improving, late and long-term effects (LLTE) affecting survivors are becoming increasingly important. It has been shown that, apart from dying from AML, suffering long-term side effects is the biggest concern of AML LTS [6]. In this context, long-term effects are persisting medical conditions that occurred during the acute treatment period as a result of the disease or its treatment, whereas late effects refer to those emerging after the end of therapy. Long-term adverse consequences of AML and its treatment may be particularly relevant in younger adults due to the larger proportion of LTS in this age group and their longer remaining lifespan. Several factors hinder gathering comprehensive data on long-term health and social outcomes, including the rareness of AML and its historically relatively low survival rates. So far, relatively little is known about LLTE caused by the disease and its treatment.

Previous studies suggested that LTS after allogeneic hematopoietic stem cell transplantation (alloHSCT) report lower quality of life than LTS treated with chemotherapy alone [7], while another study reported older age, comorbidities, and treatment with alloHSCT increased the risk for poor global health status [8]. Fatigue, mental health concerns, infections, infertility and sexual dysfunction, financial toxicity, and fear of cancer recurrence are amongst the major challenges faced by AML LTS [9]. However, previous studies in the field often had limitations such as being restricted to specific subgroups defined by age or treatment modality, or also included survivors with diseases other than AML [10–13].

We therefore conducted a cross-sectional multicenter questionnaire-based study to comprehensively assess psychosocial and somatic health outcomes in a large group of AML LTS ≥ 5 years after initial diagnosis. Here, we report the psychosocial outcomes, focusing on quality of life (QoL) and life satisfaction (LS), while additional data on additional socio-economic outcomes, somatic LLTE, and clonal hematopoiesis will be presented in the future.

## Methods

### Study participants

Potential study participants were identified from completed first-line clinical trials for adult AML patients of the AMLCG study group [AMLCG 1999 [14] (NCT00266136), AMLCG 2004 [15], AMLCG 2008 [16] (NCT01382147)], as well as the AMLCG patient registry (DRKS00020816). The registry recruits patients treated in- and outside of clinical trials. Former patients were eligible for participation in our cross-sectional study if they had received a diagnosis of AML excluding acute promyelocytic leukemia, were aged ≥18 years at the time of diagnosis, were known to be alive and in clinical remission ≥5 years after initial AML diagnosis, and were able to read and understand German in order to complete the study questionnaires and provide consent. The study was approved by the ethics committee at LMU Munich (no. 17-444). All participants provided written informed consent. This study has been registered in the German Clinical Trials Registry (DRKS00023991).

### Data collection

Potential participants were contacted by mail in accordance with applicable data protection laws and received an invitation letter, a consent form, and the study questionnaires including instruments interrogating demographics, QoL (Functional Assessment of Cancer Therapy—General [FACT-G] [17] and EORTC Quality of Life C30 questionnaire [QLQ C30] [18]), health-related (hr) and general (g) LS (hrLS/gLS, Questions on Life Satisfaction^Modules^ [FLZ^M^] [19]), anxiety and depression (Hospital Anxiety and Depression Scale [HADS] [20]), fatigue (Multidimensional Fatigue Inventory [MFI] [21]), social support (Oslo Social Support Scale [OSSS] [22]), as well as socio-economic status and self-reported somatic morbidity (adapted from German national health survey GEDA 2012 [23]). Responding participants also received a health questionnaire to be completed by their treating physicians. Detailed information about the survey instruments is provided in Supplementary Table S1. Scores for standardized instruments were computed according to the respective manual, including the handling of missing values.

### Endpoints

The primary objective of our study was to compare QoL (measured by FACT-G) and LS of AML LTS to adults without cancer. Individual scores (per participant) for these co-primary outcomes were transformed into standardized z-scores using age- and sex-stratified reference values (Supplementary Table S1). Details on secondary endpoints are presented in the Supplementary Methods [24].

### Statistical analysis

For the primary analysis, a multiple comparison testing strategy resulting in adjusted *p*-values was planned a-priori (Supplementary Fig. S1). Standardized z-scores (either for primary or exploratory analyses) were tested using non-parametric two-sided one-sample signed rank statistics. To identify factors specifically related to clinically relevant impairments in our co-primary outcomes, we categorized survivors into subgroups with and without impaired QoL or LS. Impaired QoL was defined by the established minimal clinically relevant difference of a FACT-G overall score ≥7 points below the age- and sex-matched reference cohort [25]. For hrLS and gLS, no widely accepted thresholds for clinically significant differences have been defined. Therefore, an age- and sex-matched z-score ≥1 standard deviation (SD) below the reference cohort was used to identify survivors with impaired LS. [19, 26]. Univariable and multivariable binary logistic regression models were applied to identify factors associated with impaired QoL or LS. Final multivariable models were reported after stepwise backward variable selection. All models are reported with odds ratios (OR) and 95% confidence interval.

Regarding cofactors, two sets of models for impaired QoL and LS were generated. In the first approach, we included only clinical, treatment-related and socio-demographic factors. These models deliberately did not include psychometric instruments measuring anxiety, depression, or fatigue, since their content partially overlaps with our primary outcomes, resulting in significant statistical correlation (Spearman ρ between .35 and .74, Supplementary Table S2). By excluding these variables from our initial multivariable models, we aimed to prevent these co-correlated variables from dominating the statistical models, allowing us to identify non-psychological factors associated with QoL and LS. In a second set of analyses, we added these psychological factors to the stepwise model building (for details, see Supplementary Methods).

Results of multivariable logistic regression models are reported by forest plots with odds ratios [OR], 95% CIs, and Wald test *p* values. A full description of statistical methods is provided in the Supplementary Methods. Analyses were performed in SPSS (version 28, IBM, Cary, NC, USA) and SAS software (version 9.4).

## Results

### Patient recruitment

**Fig. 1:**
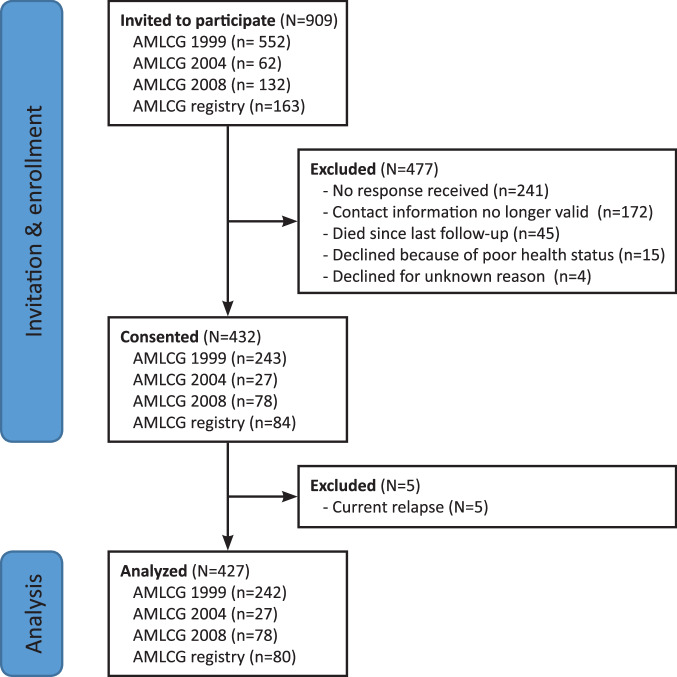
Participant flow chart. Of 909 former patients invited to participate, 423 agreed, with 427 being available for analysis.

Between December 2017 and March 2020, 909 former AML patients treated within the AMLCG study group were invited to participate in our study (Fig. 1). 432 survivors agreed to participate. Among the 477 persons who did not participate, 45 had died, 172 had moved to an unknown address, 15 declined participation for health reasons, 4 declined without giving reasons, and no response was received from 214 former patients, for a response rate of 63%. Five participants with a current AML relapse were excluded after enrollment, and the final analysis set includes data from 427 LTS (347 who received first-line treatment on AMLCG clinical trials and 80 treated upfront outside of AMLCG trials, but included in the patient registry). Supplementary Table S3 shows a comparison of clinical characteristics of participants and non-participants.

### Characteristics of AML long-term survivors

Characteristics of participating survivors are reported in Table 1. The median follow-up period after AML diagnosis was 11 years, ranging from 5 to 18.6 years. The median age at the time of study participation was 61 years, and 56% were female. Over 80% of LTS had prior de novo AML, and 72% initially had intermediate-risk cytogenetics. Sixty-three percent had undergone intensive chemotherapy (IC) followed by alloHSCT at any point during the disease course (“IC+alloHSCT”), while 37% became LTS after receiving only intensive chemotherapy without alloHSCT (“IC only”). Ninety-nine survivors (23%) became LTS after experiencing an AML relapse, 0.5 y to 18.1 y before participation. All survivors were in hematological remission at the time of study participation.

**Table 1:** Characteristics of the analyzed cohort of AML long-term survivors.

	Study participants
*N*	427
Female sex, n (%)	241 (56.4)
Age at LTS study participation [years], median (range)	61 (28–93)
Age groups, n (%)	
≤39 years	37 (8.7)
40–49 years	41 (9.6)
50–59 years	120 (28.1)
60–69 years	129 (30.2)
≥70 years	100 (23.4)
Age at AML diagnosis [years], median (range)	50 (16–80)
Time since AML diagnosis [years], median (range)	11.3 (5–18.6)
Clinical trial, n (%)	
AMLCG 1999	242 (56.7)
AMLCG 2004	27 (6.3)
AMLCG 2008	78 (18.3)
AMLCG patient registry	80 (18.7)
Relationship status, n (%)	
Single, widowed, or divorced	82 (19.2)
Married or in a relationship	345 (80.8)
Having children, n (%)	330 (77.6)
not reported	2
Number of children, median (range)	2 (1–10)
Household size, n (%)	
1 person	90 (21.1)
2 persons	255 (59.7)
≥3 persons	82 (19.2)
Highest educational degree	
University entrance qualification or higher	99 (23.3)
Other secondary education or w/o exam	325 (76.7)
Not reported, n	3
Current smoker, n (%)	58 (13.6)
AML subtype	
de novo	356 (83.4)
myelodysplasia-related	40 (9.4)
post cytotoxic therapy	31 (7.3)
MRC cytogenetic risk group at diagnosis	
favorable	61 (14.6)
intermediate	299 (71.5)
unfavorable	58 (13.8)
unknown, n	9
Treatment, n (%)	
IC + alloHSCT	269 (63.0)
IC only	158 (37.0)
AML relapse, n (%)	99 (23.2)
Years between last relapse and study participation, median (range)	9.3 (0.5–18.1)

*IC* intensive chemotherapy, *alloHSCT* allogeneic hematopoietic stem cell transplantation, *MRC* British Medical Research Council.

### Quality of life and life satisfaction in AML long-term survivors

**Fig. 2:**
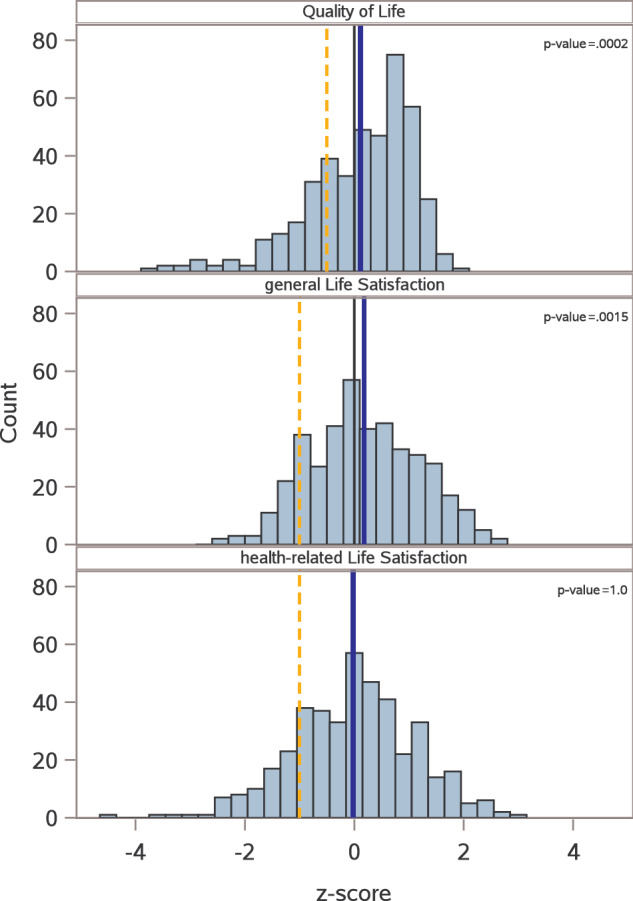
Quality of life and life satisfaction in AML long-term survivors. Histograms show, from top to bottom, AML long-term survivors’ QoL measured by the FACT-G, gLS measured by the FLZ^M^, and hrLS measured by the FLZ^M^ instrument. Data are shown as z-scores standardized against age- and sex-specific reference values from normative cohorts. Positive z scores indicate better outcomes in AML LTS compared to the reference cohort, and negative z scores indicate inferior outcomes in AML LTS vs. the reference. *P* values were adjusted through a sequentially rejective testing procedure. The blue lines indicate the means, and the orange lines indicate the thresholds used to delineate participant subsets with impaired QoL, hrLS or gLS.

AML LTS had significantly higher QoL (FACT-G mean z-score = 0.11; 95%CI: 0.01 to 0.20; adjusted p [p_adj_] = 0.0002) and higher general life satisfaction (gLS mean z = 0.18; 95%CI: 0.09–0.28; p_adj_ = 0.0015) compared to age- and sex-matched reference cohorts (Table 2 and Fig. 2), while hrLS in AML LTS was similar to the reference cohort (p_adj_ = 1.0). With respect to subscales of the FACT-G QoL questionnaire (Supplementary Table S4), AML LTS had better emotional (mean z, 0.15; 95%CI, 0.07–0.22; p_adj_ = 0.0002), social (mean z, 0.42; 95%CI, 0.35–0.50; p_adj_ = 0.0002), and functional well-being (mean z, 0.06; 95%CI, –0.03 to 0.15; p_adj_ = 0.0104) compared to the reference cohort. Physical well-being was the only subscale where AML LTS scored numerically lower than the reference cohort (mean z, –0.42; 95%CI, –0.54 to –0.30; p_adj_ = 0.0056). Of note, results on this scale are skewed (adjusted Fisher-Pearson coefficient of skewness, g = –1.36) and the median is larger than 0, indicating that this result is driven by a subset of AML LTS with impaired physical well-being, while most survivors have favorable outcomes in this domain (Supplementary Fig. S3).

**Table 2:** Primary and secondary outcomes of AML long-term survivors.

	Raw score, mean (SD)	z-scores compared to age- and sex-matched reference cohorts, mean (95%CI)^a^	*P* value
**Primary outcomes**			
Quality of Life (QoL; FACT-G)	86.0 (15.3)	0.11 (0.01 to 0.20)	0.0002^b^
General life satisfaction (gLS; FLZM)	65.3 (34.3)	0.18 (0.09 to 0.28)	0.0015^b^
Health-related life satisfaction (hrLS; FLZM)	64.6 (41.6)	–0.02 (–0.12 to 0.09)	1.0^b^
**Secondary outcomes**			
Global health status (EORTC QLQ C30)	70.6 (22.6)	0.26 (0.16 to 0.35)	<0.0001
Social support (OSSS)	10.5 (2.3)	0.24 (0.14 to 0.34)	<0.0001
Fatigue (MFI)			
General fatigue	13.4 (4.0)	–0.39 (–0.51 to –0.27)	<0.0001
Mental fatigue	15.4 (4.0)	-0.15 (–0.27 to –0.03)	0.23
Physical fatigue	14.0 (4.2)	–0.22 (–0.33 to –0.10)	0.02
Reduced activities	14.3 (4.1)	–0.18 (–0.30 to –0.07)	0.02
Reduced motivation	15.8 (3.3)	0.12 (0.02 to 0.22)	0.0001
Anxiety (HADS)	5.6 (4.0)	–0.17 (–0.29 to –0.09)	0.28
Depression (HADS)	4.4 (3.8)	0.25 (0.16 to 0.35)	<0.0001
Somatic morbidity (mFCI)	2.3 (2.0)	Not applicable	
**Socio-demographic outcomes**			
Current professional situation, n (%)			
working	192 (45.6)		
not working/ retired	229 (54.4)		
not reported, n	6		
Occupational status after AML diagnosis, n (%)			
no change	219 (52.9)		
any change	195 (47.1)		
not reported, n	13		
Financial situation after AML diagnosis, n (%)			
no change or improved	293 (69.4)		
worsened	129 (30.6)		
not reported, n	5		
Recognized disability, n (%)			
yes (any degree)	265 (63.4)		
no	141 (33.7)		
“I don’t know”	12 (2.9)		
not reported, n	9		
Among survivors with any disability:			
Degree of disability^c^, mean (95%CI)	61.8 (58.6–65.0)		
not reported, n	7		

*SD* standard deviation, *CI* confidence interval.

^a^z-scores were standardized against age- and sex-specific reference values from normative cohorts (see Supplementary Table S1). Positive z**-**scores indicate better outcomes in AML LTS compared to the reference cohort, and negative z**-**scores indicate inferior outcomes in AML LTS vs. the reference.

b: *P* values for the primary outcomes were adjusted using a sequentially rejective testing procedure.

^c^In Germany, disability is rated on a scale from 20 to 100, with a score of 50 or higher recognized as severe disability.

### Secondary outcomes

Using the EORTC QLQ C30 questionnaire, AML survivors had a better overall QoL (global health status, scale QL2) compared to the reference cohort (mean z, 0.26; 95%CI, 0.16–0.35; *p* value by signed rank test [p_sr_]<0.0001), mirroring the results of the FACT-G overall score. Survivors reported lower scores in social (mean z, -0.52; 95%CI, –0.65 to –0.40; [p_sr_]<0.001), emotional (mean z, –0.25; 95%CI, –0.36 to –0.14; [p_sr_]<0.001) and cognitive functioning subscores (mean z, –0.47; 95%CI, –0.61 to –0.34; [p_sr_]<0.001), but did not differ significantly in physical or role functioning. Additionally, AML LTS scored significantly worse on the constipation, insomnia, nausea/vomiting, fatigue and financial difficulties, but better on the appetite loss and pain symptom scales (Table 3). Using the OSSS, AML survivors had better social support (mean z-score, 0.24; 95%CI, 0.14 to 0.34; p_sr_ < 0.0001) compared to the reference cohort. Fatigue levels measured on the MFI general, physical and activity-related scales were higher in AML LTS than in the reference cohort (general fatigue: mean z, –0.39; 95%CI, –0.51 to –0.27; p_sr_ < 0.0001; physical fatigue: mean z, –0.22; 95%CI, –0.33 to –0.10; p_sr_ = 0.02, reduced activities: mean z, –0.18; 95%CI, –0.30 to –0.07; p_sr_ = 0.02;), while AML LTS scored favorably on the ‘reduced motivation’ scale (mean z, 0.12; 95%CI, 0.02–0.22; p_sr_ = 0.0001). No differences between AML LTS and reference were detected for mental fatigue (mean z, –0.15; 95%CI, –0.27 to –0.03; p_sr_ = 0.23). Depression scores, on average, were also better in AML LTS than in the reference cohort (mean z, 0.25; 95%CI, 0.16–0.35; p_sr_ < 0.0001), while there was no difference for anxiety scores (mean z, –0.17; 95%CI, –0.29 to –0.09; p_sr_ = 0.28). Of note, for these secondary outcomes, distributions were all skewed with a long tail towards worse scores (skewness g < 0), again indicating that while most AML LTS had favorable outcomes, a substantial subset of survivors had impairments in each domain (Supplementary Fig. S4).

**Table 3:** Quality of Life (measured with the EORTC QLQ C30) in comparison to general population norms.

	*N*	Raw score, mean (SD)	Relevant impairment (z <-1) N (%)	z-scores compared to age- and sex-matched reference cohorts, mean (95%CI)	*P* value^b^
**Overall Score**					
Global health status (EORTC QLQ C30)	426	70.6 (22.64)	50 (11.74)	0.26 (0.16 to 0.35)	<0.0001
**Functioning Scales**					
Social functioning	420	71.98 (32.82)	132 (31.34)	–0.52 (–0.65 to –0.40)	<0.0001
Cognitive functioning	424	77.12 (26.32)	125 (29.48)	–0.47 (–0.61 to –0.34)	<0.0001
Emotional functioning	425	70.10 (26.17)	110 (25.88)	–0.25 (–0.36 to –0.14)	0.003
Role functioning	419	72.04 (31.44)	103 (24.58)	--0.24 (–0.35 to –0.13)	0.058
Physical functioning	426	79.81 (21.21)	69 (16.20)	–0.02 (–0.11 to 0.08)	0.054
**Symptom Scales**					
Financial difficulties	423	20.17 (32.17)	110 (26.00)	–0.44 (–0.57 to –0.30)	0.012
Constipation	425	8.78 (21.26)	70 (16.47)	–0.02 (–0.12 to 0.08)^a^	<0.0001
Diarrhea	426	11.58 (21.61)	71 (16.67)	–0.15 (–0.26 to –0.04)^a^	0.052
Appetite Loss	425	8.31 (19.66)	63 (14.82)	0.01 (–0.08 to 0.10)^a^	<0.0001
Insomnia	424	34.67 (33.07)	92 (21.70)	–0.12 (–0.22 to –0.03)	0.026
Dyspnea	423	27.27 (32.25)	109 (25.77)	–0.21 (–0.32 to –0.10)	0.226
Pain	431	25.49 (30.31)	56 (13.30)	0.16 (0.06 to 0.25)	<0.0001
Nausea/Vomiting	424	4.36 (12.50)	38 (8.96)	–0.03 (–0.13 to 0.08)^a^	<0.0001
Fatigue	417	32.25 (26.91)	96 (23.02)	–0.14 (–0.24 to –0.05)	0.043

*SD* standard deviation, *CI* confidence interval.

Positive z**-**scores indicate better outcomes in AML LTS compared to the reference cohort, and negative z**-**scores indicate inferior outcomes in AML LTS vs. the reference Consequently, higher z-scores indicating less symptom burden.

^a^Skewness of the underlying distribution below –2.0.

^b^*p* values correspond to non-parametric Wilcoxon test and might result in inconsistent results compared to symmetric 95%CI presented in the neighboring column.

AML survivors had an average burden of somatic morbidities, measured on the FCI scale, of 2.3 points (95% CI: 2.1–2.5). Almost half of AML survivors (47.1%) reported a change in their occupational situation, and 30.6% reported a worsening of their financial situation associated with their disease or its treatment. Details on occupational and financial outcomes are presented in the Supplementary Results. Finally, 63.4% of AML LTS reported an officially recognized disability. Among those, the average degree of disability according to the German legal definition, on a scale of 20 to 100, was 62 (95%CI: 58.6–65.0).

### Identification of factors associated with impaired quality of life and life satisfaction

We identified subsets of AML survivors with clinically relevant impairment of QoL or LS, defined as a 7-point difference on the FACT-G score compared to an age- and sex-stratified reference cohort, or a gLS/hLS z-score more than –1 SD below the reference cohort. Overall, 27% (117/427) of AML LTS showed impaired QoL, while 14% (57/414) and 17% (74/422) reported impaired gLS and hrLS, respectively. Supplementary Fig. S2 shows score distributions of each primary outcome according to age group at participation, sex, AML type, prior relapse, and therapy. To identify factors associated with impaired QoL or LS ≥ 5 years after an AML diagnosis, we fitted multivariable logistic regression models, including potentially relevant variables identified in univariate analysis (Supplementary Tables S5–S7).

#### Models including clinical, treatment-related, and socio-demographic factors

**Fig. 3:**
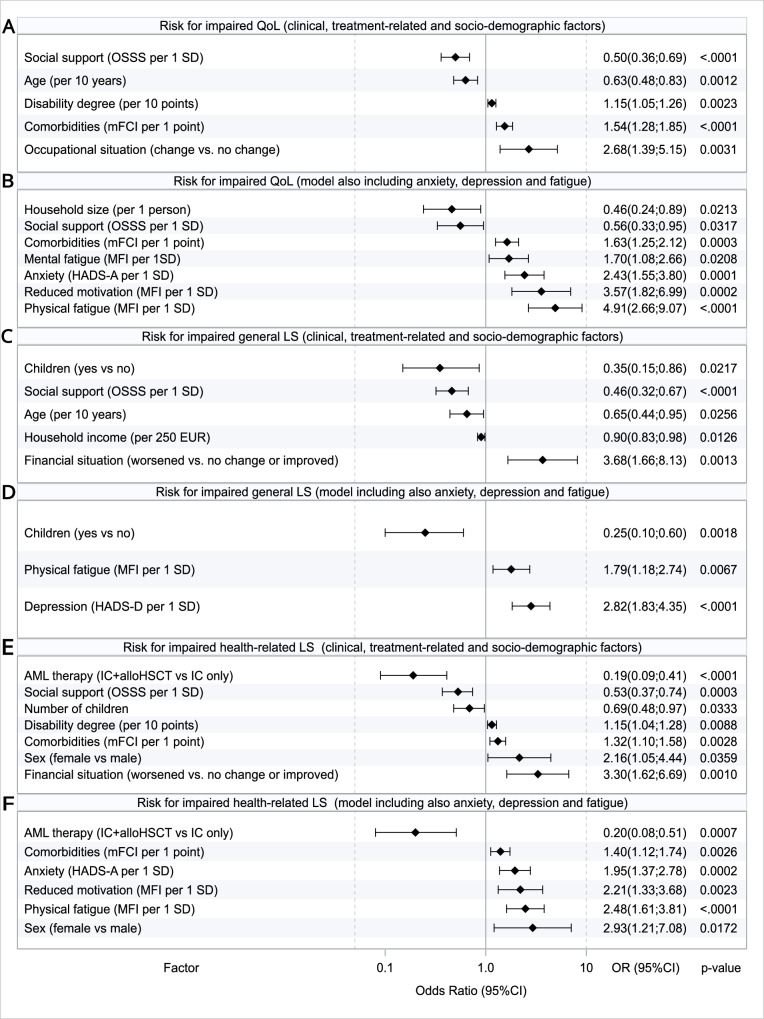
Forest plots of final multivariable logistic regression models for impaired QoL and LS after backward variable selection. Forrest Plots show, the final models of associated factors, (**A**) first considering clinical, treatment-related and socio-demographic factors, followed by the model (**B**) also considering anxiety, depression and fatigue for Qol (measured with the FACT-G). Next both models for gLS are shown (**C**, **D**), followed by both for hrLS (**E**, **F**), respectively. Note: Age is “Age at participation” in our cross-sectional study. QoL quality of life, LS life satisfaction, SD standard deviation, OR odds ratio, CI confidence interval, IC intensive chemotherapy, alloHSCT allogeneic hematopoietic stem cell transplantation.

The factor most closely associated with impaired QoL was a change in occupational situation due to AML (e.g. inability to work at all or having to reduce working hours or switch careers), resulting in a 2.7-fold increased risk of impaired QoL compared to survivors with no change (Fig. 3). With respect to life satisfaction, the factors most closely associated with impaired gLS were worsened financial situation (3.7-fold risk increase) and not having children (2.9-fold increase). Notably, treatment modality (IC only vs. IC+alloHSCT), prior AML relapse, or disease type (de novo vs. secondary AML) were not associated with QoL or gLS in these multivariable analyses. Conversely, the strongest factors for impaired hrLS were treatment with IC only as opposed to IC+alloHSCT (5.3-fold risk increase), a worsened financial situation due to AML (3.3-fold risk increase) and female sex (2.2-fold increase).

#### Models additionally considering anxiety, depression and fatigue

In the extended models also considering psycho-social variables, we identified physical and mental fatigue, reduced motivation, and anxiety as factors closely associated with impaired QoL. With respect to gLS, physical fatigue and depression closely associated with impaired life satisfaction. Finally, physical fatigue and reduced motivation as well as anxiety associated with impaired hrLS (Fig. 3).

**Fig. 4:**
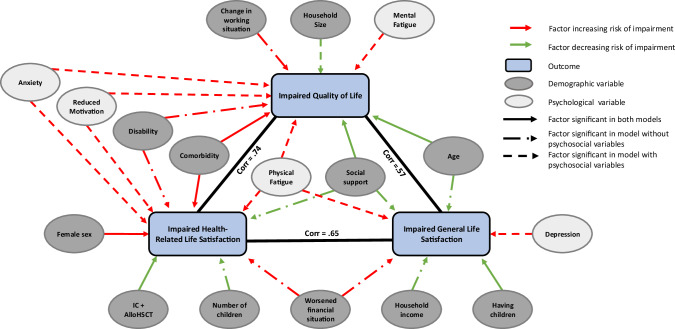
Network diagram impact for impaired QoL or LS. The network diagram shows the relationships between the three primary outcomes and the direction of association for factors contained in the final models. IC intensive chemotherapy, alloHSCT allogeneic hematopoietic stem cell transplantation, Corr correlation.

Figure 4 provides an overview of the factors associated with each of our co-primary outcome measures in either of the two model-building approaches. Physical fatigue as well as social support emerged as the only factors associated with all primary outcomes. Furthermore, QoL and hrLS were impacted by a group of shared factors including somatic comorbidity, physical disability, anxiety and reduced motivation, whereas a distinct set of non-treatment related variables were identified as associated with gLS.

## Discussion

Our study, representing one of the largest cohorts of AML long-term survivors studied so far, reveals that QoL of AML LTS five or more years after the initial diagnosis, on average, reaches levels comparable to adults without a history of cancer. Similar results were found in a longitudinal study that examined QoL in intensively treated AML patients for three years after their initial diagnosis, with almost 80 percent reporting “normal” QoL [27]. Since other studies with shorter follow-up reported impaired QoL [7], this may indicate that QoL of survivors recovers over several years after diagnosis and treatment of AML. However, no association between time since diagnosis beyond 5 years and QoL was identified in our study, similar to a prior large study including various blood cancers [28].

Even though many AML survivors reported good QoL, one in four long-term survivors still experience meaningful impairment even ≥5 years after initial diagnosis. Like overall QoL, most secondary outcomes evaluated in our study also showed a skewed distribution (Supplementary Fig. S3), indicating that a substantial minority of survivors have lasting impairment and might benefit from additional support. We were able to identify factors associated with long-term impairment of QoL and health satisfaction, including a change in occupational status, a higher degree of disability, higher number of comorbidities, and higher fatigue and anxiety levels. On the other hand, we also identified protective factors including older age, better social support and a bigger household size, which in part align with previous research [8]. Likewise, a prior, single-center study of 5-year survivors of various hematological cancers identified medical comorbidity, psychological distress, lower social support, and high levels of fatigue and functional impairment as factors associated with a stronger negative impact of cancer on subsequent quality of life [29]. Of note, in this study, acute leukemia survivors scored significantly higher on the scales measuring positive impact of cancer (e.g., on the positive self-evaluation and health awareness scales of the Impact of Cancer Questionnaire) compared to survivors of other entities, in agreement with the favorable overall outcomes seen in our cohort.

While some socio-demographic factors for impaired QoL such as younger age and lower levels of social support cannot be directly influenced by the medical care team, they identify individuals at a greater risk for impairment in QoL who may benefit from more intensive psychosocial support. Even though a significant proportion of cancer survivors reportedly show resilience or post-traumatic growth, younger age has been reported as a risk factor for adverse survivorship outcomes across cancer entities [30]. Complex interactions of various factors, such as being diagnosed during particularly sensitive life stages (e.g. while establishing financial security, developing an own identity, or raising a family), as well as negative peer comparisons due to impacts of the disease and its treatment may contribute to this effect. Additionally, some risk factors such as changes in occupational situation, which had a strong impact on QoL in our study, may particularly affect younger survivors. Another large German study showed that more than a third of former blood cancer patients had not returned to work ≥3 years (median, 9 years) after their initial diagnosis [28]. Consequently, QoL might be positively impacted through targeted occupational programs addressing these aspects.

Our study also evaluated life satisfaction of survivors. In contrast to QoL, LS captures a person’s individual perception of how far their life quality matches a self-imposed standard [31]. LS has been rarely addressed in prior research on AML survivors [11]. We were able to differentiate between gLS and hrLS, and identified specific factors associated with favorable or unfavorable outcomes for each domain. Overall, life satisfaction was less commonly impaired than QoL, indicating that some survivors were satisfied with their current life situation even in the presence of a significant symptom load captured by the QoL instruments. This finding, as well as the mostly favorable QoL outcomes in our cohort, might be explained by “response shift”, which describes the phenomenon that the meaning of some constructs and items in patient-centered outcome measures is time dependent, and patients may interpret them differently as they go through new life experiences [32]. Potential mechanisms of response shifts include recalibrating (i.e., survivors may have adopted a novel reference frame due to the experiences made during their disease), reprioritizing (i.e., a change in the importance of different components in the target), reconceptualizing (i.e. redefining the construct altogether) or a combination of these. This points to life satisfaction being an important outcome in survivorship research.

Figure 4 shows a network diagram integrating our results on factors that positively or negatively affect QoL and LS of AML survivors. It highlights a key strength of our study: our multi-dimensional approach that captures different aspects of survivorship outcomes, as well as a broad range of potentially impacting factors. Overall, physical fatigue emerged as the only factor associated with worsening of all three primary outcomes, while better social support was a protective factor for all three. Other factors affecting hrLS (e.g., anxiety, comorbidity burden, and physical fatigue) were also relevant for QoL, suggesting interventions addressing them could benefit both domains.

Somatic comorbidities were associated with impaired QoL and hrLS. AML survivors in our study had a mean of 2.3 comorbid conditions captured by the FCI score. In a recent study, 2-year survivors of allogeneic transplantation had a 3.8-times higher risk of severe or life-threatening conditions compared to their siblings [33], also emphasizing the importance of addressing late and long-term morbidity as an underlying cause for long-term impairment of QoL. Screening for medical comorbidities and treatment of the associated symptoms may not only help to improve QoL in AML LTS but also improve late mortality that has been observed in young AML survivors [34].

Notably, treatment modality (i.e., whether a patient had received an allogeneic transplantation or only cytotoxic chemotherapy) did not consistently associate with QoL or LS and in particular, allogeneic transplantation or a prior disease relapse did not correlate with poorer outcomes among our participants (Supplementary Table S8). Indeed, survivors who had undergone alloHSCT reported better hrLS than participants treated with chemotherapy alone. Again, response shift due to re-calibration of patients’ expectations after the experience of undergoing alloHSCT may be one factor contributing to these favorable self-reported outcomes. Alternatively, survivors suffering from severe transplant-induced LLTE, such as chronic GvHD, might have been less willing to participate in our study or may have been more likely to die before the 5-year landmark. However, such a selection bias would be unlikely to completely eliminate or even reverse any between-group differences.

While QoL and hrLS shared a subset of overlapping associated factors that may be long-term consequences of leukemia and its treatment, gLS was linked to a different subset of factors such as household income or having children, which are only partly linked to the disease. Thus, general life satisfaction seems to be only weakly affected by AML-related factors in long-term survivors.

Strengths of our study include its large cohort size, multicentric recruitment, and our multi-dimensional approach. We focused on a single entity and only included survivors in remission, since prior studies in mixed population have revealed differences in survivorship outcomes between different entities [28]. On the other hand, we included survivors across a broad age range and did not restrict our analysis to a specific treatment modality, such as allogeneic transplantation. Overall, we believe our data are representative of AML survivors in Germany. Our study also has limitations. Although we used repeated contact attempts to achieve a high response rate, we cannot fully exclude non-responder bias. However, the response rate of 63% can be considered very high for a study enrolling former patients from multiple centers 5 to 18 years after diagnosis and exceeds the previously recommended threshold of 60% for oncology patient surveys [35]. As pointed out by Nishimura et al. [36], response rates are not well-suited to assess non-responder bias. Comparing data from responders and non-responders using available baseline data (collected at diagnosis) did not reveal any severe or unexpected differences, but this only provides an indirect assessment of potential non-responder bias. Other statistical approaches to diagnose or adjust for non-response bias also have weaknesses [36]. The possibility that participants with strongly impaired QoL – or, vice versa, particularly good QoL- might have preferentially declined participation in our study therefore remains. On the other hand, despite our cohort’s particularly long follow-up, the response rate in our study compares favorably to previously reported cohorts of AML survivors (44–69%), supporting the generalizability of our findings [7, 8, 10, 11, 16, 29, 31, 33]. Due to our cross-sectional approach, we did not collect information on intra-individual trajectories of QoL and LS over time. We used the FACT-G overall score as a comprehensive and robust instrument capturing multiple domains of QoL within a single summary score as part of our primary endpoint. Available FACT-G normative data were derived from an Austrian population sample, which, although originating from a neighboring, culturally similar and German-speaking country, might have introduced bias. However, analysis of the EORTC QLQ-C30 Global Health Status score using contemporary German normative data yielded results consistent with the FACT-G summary score. Lastly, our multivariable analysis for impaired QoL and LS revealed a strong intercorrelation between psychosocial variables and the primary outcomes, which may hamper identification of other relevant associations. Further research is warranted to identify causal relationships that impact survivorship outcomes, and that might be addressed in risk-adapted survivorship care programs.

In conclusion, our study presents long-term outcomes from one of the largest cohorts of acute leukemia survivors. Patients and physicians battling this disease should be reassured by our finding that QoL and LS of many AML LTS are comparable to reference populations without leukemia. Importantly, however, a subset of survivors continues to experience impaired global QoL even ≥5 years after their initial diagnosis. Furthermore, AML LTS show persistent impairment in several specific domains of QoL, such as social functioning. Collectively, these data highlight the need to include guidance for structured follow-up care into clinical guidelines [37]. Our data identify relevant problem fields of AML survivors that should be subject of further studies, and that might be modified by targeted interventions. As many cancer survivors, and especially those with a perceived disease-related burden, are dissatisfied with their medical care [38], our data may provide a foundation to develop improved, needs-based and risk-adapted survivorship care models for AML LTS beyond the five-year mark after diagnosis.

## Supplementary information

Supplemental Material, clean version

## Data Availability

The datasets generated and/or analysed during the current study can be made available from the corresponding author on reasonable request.
