# Supplementary material for: Quality of life and life satisfaction in long-term survivors of acute myeloid leukemia
Source: Leukemia. 2025 Aug 22;39(11):2663–72. doi: 10.1038/s41375-025-02735-y (PMC12589116; doi:10.1038/s41375-025-02735-y)
Supplement: Supplementary file 1 — Supplemental Material, clean version [file 41375_2025_2735_MOESM1_ESM.pdf]

Supplementary Appendix to

Quality of Life and Life Satisfaction in AML Long-Term Survivors

Eva Telzerow, Dennis Görlich, Cristina Sauerland, Maja Rothenberg-Thurley, Anna Sophia Moret, Simon M. Krauß, Friederike H. A. Mumm, Susanne Amler, Wolfgang E. Berdel, Bernhard J. Wörmann, Utz Krug, Jan Braess, Pia Heußner, Wolfgang Hiddemann, Karsten Spiekermann, and Klaus H. Metzeler

Contents

1. Supplemental Methods 2
1.1. Computation of z-scores 2
1.2. Analysis of primary endpoints 2
1.3. Definition of dichotomized outcomes 3
1.4. Analysis of secondary endpoints 3
1.5. Exploratory data analysis and regression models 4
2. Supplementary Results: Impact on occupational and financial situation 5
3. Supplementary Tables 6
3.1. Supplementary Table S1: Survey Instruments 6
3.2. Supplementary Table S2. Spearman correlation between the different survey instruments (N=380 complete observations) 9
3.3. Supplementary Table S3. Clinical characteristics of study participants compared to potential participants who were not enrolled 10
3.4. Supplementary Table S4: Results of the primary statistical testing procedure based on the non-parametric elementary tests 11
3.5. Supplementary Table S5. Univariate and multivariable logistic regression analysis of factors associated with impaired QoL 12
3.6. Supplementary Table S6. Univariate and multivariable logistic regression results for impaired gLS 14
3.7. Supplementary Table S7. Univariate and multivariable logistic regression results for impaired hrLS 16
3.8. Supplementary Table S8. Crude analysis of the association between allogenic HSCT and prior AML relapse and impaired quality of life or life satisfaction 18
4. Supplementary Figures 19
4.1. Supplementary Figure S1. Graph of the predefined sequential testing procedure 19
4.2. Supplementary Figure S2. Distribution of QoL (FACT-G) and life satisfaction (gLS, hLS) by age group, AML type, relapse, sex and therapy 20
4.3. Supplementary Figure S3: Density plots of outcomes on FACT-G subscales 21
4.4. Supplementary Figure S4: Density plots of secondary outcomes 22
5. Supplementary References 23

1. Supplementary Methods

1.1. Computation of z-scores

Individual participants' z-scores were calculated by standardization:

$$z_i = \frac{X_i^D - M^{D(age(i),sex(i))}}{SD^{D(age(i),sex(i))}}$$

With X_i^D as raw score of the instrument D of patient i; $M^{D(age(i),sex(i))}$ as mean of measure D in the age-and sex- stratified subgroup of patient i; $SD^{D(age(i),sex(i))}$ as standard deviation of measure D in the age-and sex- stratified subgroup of patient i; using age- and sex-stratified population norms (see Supplementary Table S1). All z-scores were calibrated to represent improvement over the reference population by z >0. In particular, this implies that measures which increase with worsening symptoms (HADS and MFI) were computed as shown above and then inverted (z multiplied with -1).

1.2. Analysis of primary endpoints

Regarding our primary study endpoints, we test standardized z-scores for QoL (FACT-G), hrLS and gLS (FLZ^M) within a multiple comparison procedure to control the multiple significance level at 5%. The statistical tests were embedded in a graph-based sequentially rejective testing procedure (SRTP) prespecified before data collection. The mathematical basis of the chosen graph-based methodology was published by Bretz et al. ((1, 2)). Bretz et al. defined the procedure and proved that this method controls the family-wise error rate in the strong sense.

For the primary analysis all null-hypotheses are of form:

$$H_0^D: mean(z^D) = 0,$$

for each tested instrument $D \in \{QoL_{FACT-G}, QoL_{FACT-G\ PWB}, QoL_{FACT-G\ EWB}, QoL_{FACT-G\ FWB}, QoL_{FACT-G\ SWB}, gLS, hrLS\}$

(Abbrev: PWB – Physical well being; EWB – Emotional well being; FWB – Functional well being; SWB – social well being; gLS – general life-satisfaction; hrLS – health related life-satisfaction).

We designed the graph of null-hypotheses to test the total scores of all three primary outcomes first and allows, in case of significant results, to use the significance level effectively also for the subsequent tests on the remaining hypotheses in a stepwise manner (Supplementary Figure S1). Adjusted p-values (p_{adj}) were computed from the procedure. Elementary hypotheses were tested by two-sided non-parametric Wilcoxon signed-rank tests. By this we deviated from the a-priori planned t-test approach, because of stronger than expected skewness of the z-score distributions.

1.3. Definition of dichotomized outcomes

We defined impairment on the three primary outcomes QoL (FACT-G), general life-satisfaction (gLS) and health-related life-satisfaction (hrLS) by cutoff values. For impairment of QoL we defined by a 7-point difference on the FACT-G score compared to age- and sex-stratified reference values.(3) The 7 points are considered clinically relevant(4). Since no clinically relevant cut off score are available for gLS and hrLS, scores were dichotomized for impairment at scores of z < -1 SD. Threshold raw score values for each instrument can be found in Supplementary Table S9.

1.4. Analysis of secondary endpoints

Secondary endpoints included QoL measured by the EORTC QLQ C30, social support, fatigue, anxiety and depression symptoms, officially recognized disability, and occupational and financial status (Supplementary Table 1). Cumulative burden of comorbidities was graded according to a modified version of the functional comorbidity index (mFCI), based on self-reported and physician-reported diagnoses.

Functional Comorbidity Index (FCI) (5)	modified FCI
Arthritis	Arthritis
Osteoporosis	Osteoporosis
Asthma	Asthma or COPD/ARDS/emphysema (combined into one category)
Chronic obstructive pulmonary disease (COPD), acquired respiratory distress syndrome (ARDS) or emphysema	
Angina	Angina
Congestive heart failure	Congestive heart failure
Heart attack	Heart attack
Neurological disease	Neurological disease
Stroke or TIA	Stroke or TIA
Peripheral vascular disease	*Not included*
Diabetes Type I or II	Diabetes Type I or II
Upper gastrointestinal diseases	Upper gastrointestinal diseases
Depression	Depression or anxiety or panic disorder (combined into one category)
Anxiety or panic disorder	
Visual impairment	Visual impairment
Hearing impairment	Hearing impairment
Degenerative disc disease	Degenerative disc disease
Obesity	Obesity

Z-scores of secondary endpoints were computed according to the methodology described above. mFCI was excluded from z-standardization, since no published reference values can be applied. Non-parametric methodology (signed rank tests to test $H_0=0$) were used due to the skewness of distributions.

1.5. Exploratory data analysis and regression models

In further exploratory analyses, we identified factors influencing impaired Qol and LS using the following approach: First, univariate binary logistic regression models were used to identify potential influencing factors. We computed multivariable binary logistic regression models including factors with univariate p-value ≤ 0.2 including a backward variable selection. Results of the final multivariable model are reported as odds ratios (OR) and 95% CI.

To identify risk factors for QoL and LS candidate variables were individually tested in univariate models. Potential risk factors resulting in univariate p-values of ≤0.2 were include in the backward selection process to compute the final model. The chosen cutoff value of 0.2 allowed us to preselect factors into the analysis which might be influential, but did not reach significance in the univariate analysis. By this we start the backward selection with a model that contains relevant information on the outcome. Full details for all models were reported in Supplementary Tables 3.5 – 3.7. Figure 4 in the main texts shows a summary network diagram displaying the associations reported by the final statistical models for impaired QoL, gLS and hrLS.

2. Supplementary Results: Impact on occupational and financial situation

We specifically collected information about the impact of AML diagnosis and treatment on survivors' occupational and financial situation. Multivariable logistic regression models showed that occupational and financial situation associate with quality of life and life satisfaction in AML LTS. Here, we present more detailed information on occupational and financial variables. Among 414 AML LTS with available data, 52.9% (219) reported no change in occupation status associated with their disease. Overall, the most frequent disease-related change was early retirement in 24.2% (100/414)]. Female survivors more often reported a change in occupation situation than males (53.0% vs 39.6%, $p=0.0044$), mainly due to an increased frequency of reducing working hours (14.7% vs 7.1%). A disease-related change in occupational status was less common in older survivors (>= 62 years at survey participation) (39.4% vs 53.8% for younger survivors, $p<0.0001$), most likely due to the higher proportion of participants who retired independently of their disease. Compared to younger participants, older survivors were less likely to reduce their working hours (5.2% vs 16.7%) and more likely to retire (28.0% vs 20.8%).

Data on changes to their financial situation was available for 422 survivors. Two thirds (66.8%) reported no change, 30.6% reported a worsened financial situation, and 2.6% reported an improved financial situation. Financial outcomes did not differ significantly between male and female survivors. Younger survivors more often reported a worsening of their financial situation than older participants (36.8% vs 23.0%, $p=.0002$).

3. Supplementary Tables

3.1. Supplementary Table S1: Survey Instruments

Instrument	Content	Source of Reference Data	Number of Items	Number of Subscales	Item Range	Summary Score Range
**Primary Outcomes**						
**FACT-G(6)**<br>Functional Assessment of Cancer Therapy – General	Quality of life instrument, contains four subscales (physical well-being (PWB), emotional well-being (EWB), functional well-being (FWB) and social well-being (SWB))	Holzner et al., 2004(3)	27	4	0 to 4	Total score:<br>0 to 108<br>PWB, FWB, SWB:<br>0 to 28<br>EWB: 0 to 24
**FLZ^{M} (7)**<br>Questionnaire for life satisfaction Munich	Life satisfaction instrument, containing two scales: heath related life satisfaction (hrLS) and general life satisfaction (gLS). Both scales weigh the satisfaction of the individual items with the respective importance of the items. Higher scores mean higher life satisfaction.	Daig et al., 2009(8)<br><br>Manual of the FLZ^{M} questionnaire(7)	33	2	1 to 5	-96 to 160 for each subscale
**Secondary Outcomes**						
**EORTC QLQ C30(9)**<br>EORTC Quality of Life Questionnaire C30	QoL instrument, one scale for global health status, five functional scales (physical functioning, role functioning, emotional functioning, cognitive functioning, social functioning) and nine symptom scales	Nolte et al, 2020(10)	30	1 global scale<br>5 functioning scales<br>9 symptom scales	1 to 4<br>(items 1-28)<br>1 to 7<br>(items 29,30)	0 to 100 for each subscale

Instrument	Content	Source of Reference Data	Number of Items	Number of Subscales	Item Range	Summary Score Range
	(fatigue, nausea and vomiting, pain, dyspnea, insomnia, appetite loss, constipation, diarrhea, financial difficulties). Higher functioning scores representing higher functioning, higher scores on symptom scales suggesting higher symptom burden.					
**HADS(11, 12)** Hospital Anxiety and Depression Scale	Instrument measuring anxiety and depression symptoms, resulting in a summary score for each. Higher scores represent higher depression/anxiety symptom burden.	Hinz et al., 2001(13)	14	2	1 to 4	0 to 21 for each subscale
**MFI(14)** Multidimensional fatigue inventory	Fatigue instrument with five scales (general fatigue, physical fatigue, mental fatigue, motivation, activities). Higher scores represent higher burden of fatigue.	Schwarz et al., 2003 (15)	20	5	1 to 5	4 to 20 for each subscale
**OSSS(16)** Oslo Social Support Scale	Brief instrument assessing social support. A higher summary score represents high social support.	Kocalevent et al., 2018(17)	3	1	1 to 4 (Item 1) 1 to 5 (Items 2,3)	3 to 14
**Comorbidity Measure**	Self-assessment of 17 comorbidities	Adapted from GEDA 2012 survey(18)	21	-	No, yes, yes in the past year	-

Instrument	Content	Source of Reference Data	Number of Items	Number of Subscales	Item Range	Summary Score Range
					Two items about medications<br>Two items detailing potential other cancer diagnosis	
**Sociodemographic, disease and treatment related questions**	created by the study team	-	-	-	-	-
**Assessment of occupational and financial situation**	Self-assessment containing questions on working and financial situation	Adapted from GEDA 2012 survey(18)	-	-	-	-
**Modified Functional Comorbidity Index(5)**	Using information provided by the participants, their respective general practitioner's and by available medical records, two researches independently scored the comorbidity as present or not. The original index contains 18 items, due to our data collection method and combining different data sources, we were not able to replicate the index exactly, therefore resulting in 15 items.	-	15	1	0 to 1	0 to 15

3.2. Supplementary Table S2. Spearman correlation between the different survey instruments (N=380 complete observations)

	General Life Satisfaction[a]	Health-related Life Satisfaction[a]	Quality of Life[b]	Quality of Life[c]	Depression[d]	Anxiety[d]	General fatigue[e]	Physical fatigue[e]	Mental fatigue[e]	Reduced activities[e]	Reduced motivation[e]
**General Life Satisfaction[a]**		0.66	0.65	0.56	-0.56	-0.31	-0.42	-0.50	-0.34	-0.45	-0.52
**Health-related Life Satisfaction[a]**	0.66		0.75	0.68	-0.68	-0.46	-0.58	-0.69	-0.46	-0.60	-0.66
**Quality of life[b]**	0.67	0.75		0.73	-0.77	-0.58	-0.70	-0.75	-0.55	-0.68	-0.67
**Quality of Life[c]**	0.54	0.67	0.71		-0.61	-0.40	-0.62	-0.73	-0.40	-0.57	-0.55
**Depression[d]**	0.55	0.66	0.75	0.58		0.53	0.59	0.65	0.48	0.63	0.67
**Anxiety[d]**	0.33	0.50	0.59	0.40	0.56		0.46	0.34	0.45	0.26	0.33
**General fatigue[e]**	0.43	0.58	0.69	0.57	0.62	0.49		0.72	0.53	0.66	0.54
**Physical fatigue[e]**	0.50	0.68	0.75	0.69	0.66	0.40	0.77		0.44	0.76	0.64
**Mental fatigue[e]**	0.36	0.48	0.56	0.39	0.50	0.47	0.58	0.50		0.52	0.51
**Reduced activities[e]**	0.44	0.60	0.69	0.56	0.64	0.32	0.70	0.76	0.56		0.69
**Reduced motivation[e]**	0.51	0.65	0.67	0.54	0.66	0.38	0.57	0.64	0.54	0.68	

Footnotes: Data are shown for N=380 complete observations. Upper-right triangle: correlation among raw score values. All p-values < 0.0001. Color indicates direction: blue, positive correlation; red: negative correlation. Lower-left triangle: correlation among z-standardized values. All z-values were computed to indicate better outcomes with positive signs, while negative z-values always indicate worse than normal/healthy scores. a) FLZM b) FACT -G c) EORTC QLQ C30 d) HADS e) MFI

3.3. Supplementary Table S3. Clinical characteristics of study participants compared to potential participants who were not enrolled

	Invited potential participants N=909	Enrolled N=432	No response or no consent N=477	p-value
Female sex, n(%)[1]	488 (53.7)	243 (56.4)	245 (51.4)	.14 [a]
Age at LTS study invitation [years], median (range)	62 (25-93)	61 (28-93)	63 (25-92)	.04[b]
Age groups at LTS study invitation, n (%)[1]				<.0001[a]
≤39 years	73 (8.0)	33 (7.6)	40 (8.4)	
40-49 years	94 (10.3)	39 (9.0)	55 (11.5)	
50-59 years	217 (23.8)	115 (26.6)	102 (21.4)	
60-69 years	243 (26.7)	139 (32.2)	104 (21.8)	
≥70 years	282 (31.2)	106 (24.5)	176 (36.9)	
Age at diagnosis, years Median (range)	50 (16-85)	50 (16-80)	50 (16-85)	.24 [b]
Time since diagnosis, years Median (Range)	11.8 (5-18.8)	11.1 (5-18.4)	12.3 (5-18.8)	.0015 [c]
Clinical trial, n(%)[1]				.01[a]
AMLCG 1999	552	243 (56.3)	309 (64.8)	
AMLCG 2004	62	27 (6.3)	35 (7.3)	
AMLCG 2008	132	78 (18.1)	54 (11.3)	
AMLCG patient registry	163	84 (19.4)	79 (16.6)	
AML type, n(%)[1]				.33 [a]
de novo	756 (85.1)	360 (83.3)	396 (86.8)	
sAML	76 (8.6)	42 (9.7)	34 (7.5)	
tAML	56 (6.3)	30 (6.9)	26 (5.7)	
unknown	21	0	21	
Cytogenetic risk, n(%)[1]				.06 [a]
low	135 (15.4)	60 (14.3)	75 (16.5)	
intermediate	634 (72.5)	299 (71.2)	335 (73.8)	
unfavorable	106 (12.1)	62 (14.7)	44 (9.7)	
unknown	34	11	23	
NPM1 mutation, n(%)	283 (50.5)	141 (49.8)	142 (51.1)	.76 [a]
unknown, n	348	283	278	
FLT3 mutation [e], n(%)	132 (22.0)	68 (22.7)	64 (21.3)	.68 [a]
unknown, n	308	132	176	
Treatment, n(%)[1]				<.0001 [a]
IC + alloHSCT	460 (50.1)	262 (60.7)	198 (41.5)	
IC only	449 (49.4)	170 (39.4)	279 (58.5)	
Prior relapse, n(%)[1]	194 (21.3)	100 (23.2)	94 (19.7)	.21 [a]

Footnotes: 1: Percentages within responders / non-responders (column percentage). [a] Chi-Squared test. [b] t-test with Satterthwaite degrees of freedom due to unequal variances. [c] Non-parametric Mann-Whitney U test. [e] *FLT3* ITD or TKD mutation.

3.4. Supplementary Table S4: Results of the primary statistical testing procedure based on the non-parametric elementary tests

Instrument and subscale	Mean z-scores (95%CI)	Median (Q1;Q3)	Raw p-value	Adjusted p value (SRTP)
**Quality of life Sum Score** (FACT-G)	0.11 (0.01 to 0.20)	0.30 (-0.52;0.83)	<.0001	.0002
**General life satisfaction** (FLZ^{M})	0.18 (0.09 to 0.28)	0.13 (-0.53;0.89)	.0005	.0015
**Health-related life satisfaction** (FLZ^{M})	-0.02 (-0.12 to 0.09)	0.01 (-0.77;0.64)	.99	1
**Emotional Well-Being** (FACT-G)	0.15 (0.07 to 0.22)	0.35 (-0.35;0.77)	<.0001	.0002
**Physical Well-Being** (FACT-G)	-0.42 (-0.54 to -0.30)	0.13 (-1.12;0.53)	.0009	.0056
**Social Well-Being** (FACT-G)	0.42 (0.35 to 0.50)	0.60 (0.03;0.94)	<.0001	.0002
**Functional Well-Being** (FACT-G)	0.06 (-0.03 to 0.15)	0.20 (-0.48;0.80)	.0032	.0104

Abbreviations: SRTP, sequentially rejective testing procedure
Footnotes: Non-parametric analysis was chosen due to the skewed distribution of z-scores.

3.5. Supplementary Table S5. Univariate and multivariable logistic regression analysis of factors associated with impaired QoL

Factor	Univariate models			Entered into model building	Final multivariable model after backward selection		Final model with depression, anxiety and fatigue variables available for selection	
	N	OR (95%CI)	p		OR (95%CI)	p	OR (95%CI)	p
Sex (female vs male)	427	0.91 (0.59 to 1.39)	.66	N				
Age at study participation, per 1 year	427	0.99 (0.97 to 1.00)	.12	Y	0.96 (0.93 to 0.98)	.0012	Not selected	
Age at diagnosis, per 1 year	427	0.99 (0.98 to 1.01)	.24	N				
In a relationship (yes vs no)[†]	427	0.72 (0.43 to 1.21)	.21	N				
Children (yes vs no)	427	0.66 (0.41 to 1.08)	.10	Y	Not selected		Not selected	
Number of children, per 1	425	0.84 (0.70 to 1.01)	.07	N				
Currently smoking (yes vs no)	427	1.48 (0.82 to 2.66)	.20	Y	Not selected		Not selected	
Education (University entrance qualification or higher vs other)	412	1.53 (0.90 to 2.63)	.12	Y	Not selected		Not selected	
Weekly work hours, per 1 hour	398	0.99 (0.97 to 1.00)	.04	Y	Not selected		Not selected	
Occupational situation (changed vs no change)	414	3.14 (1.99 to 4.96)	<.0001	Y	2.68 (1.39 to 5.15)	.0031	Not selected	
Employment status (yes vs no)	421	0.64 (0.41 to 1.00)	.05	Y	Not selected		Not selected	
Household size, per 1 person	427	0.77 (0.59 to 1.00)	.05	Y	Not selected		0.46 (0.24 to 0.89)	0.02
Household income, per 250 EUR	407	0.94 (0.91 to 0.98)	.002	Y	Not selected		Not selected	
Per capita income, per 250 EUR	407	0.92 (0.85 to 0.99)	.02	N(i)				
Financial situation (worsened vs. no change or improved)	422	3.76 (2.39 to 5.92)	<.0001	Y	Not selected		Not selected	
Disability (yes vs no)	399	3.16 (1.85 to 5.41)	<.0001	N				
Disability degree, per 10 points	399	1.16 (1.09 to 1.23)	<.0001	Y	1.15 (1.05 to 1.26)	.0023	Not selected	
Relapse (yes vs no)	427	0.70 (0.41 to 1.19)	.19	Y	Not selected		Not selected	
Treatment (IC+alloHSCT vs IC)	427	1.02 (0.65 to 1.58)	.95	N				
AML type (*de novo* vs s- or t-AML)	427	0.81 (0.47 to 1.41)	.46	N				

Factor	Univariate models			Entered into model building	Final multivariable model after backward selection		Final model with depression, anxiety and fatigue variables available for selection	
	N	OR (95%CI)	p		OR (95%CI)	p	OR (95%CI)	p
Time since diagnosis, per 1 year	427	0.96 (0.91 to 1.02)	.18	Y	Not selected		Not selected	
Time since last therapy, per 1 year	291	0.97 (0.91 to 1.03)	.34	N				
Social support (z-score, per 1 SD)	423	0.47 (0.39 to 0.59)	<.0001	Y	0.50 (0.36 to 0.69)	<.0001	0.56 (0.33 to 0.95)	0.03
Modified Functional Comorbidity Index, per point	378	1.42 (1.23 to 1.60)	<.0001	Y	1.54 (1.28 to 1.85)	<.0001	1.63 (1.25 to 2.12)	0.0001
Additional psychometric variables (Fatigue, anxiety and depression)								
General fatigue (MFI-general, z-score, per 1 SD)	419	0.31 (0.24 to 0.40)	<.0001				Not selected	
Reduced Activities (MFI-activities, z-score, per 1 SD)	418	0.27 (0.20 to 0.35)	<.0001				Not selected	
Physical fatigue (MFI-physical, z-score, per 1 SD)	420	0.21 (0.16 to 0.30)	<.0001				0.20 (0.11 to 0.38)	<.0001
Mental fatigue (MFI-mental, z-score, per 1 SD)	424	0.40 (0.32 to 0.49)	<.0001				0.59 (0.38 to 0.92)	.02
Reduced motivation (MFI-motivation, z-score, per 1 SD)	420	0.20 (0.14 to 0.28)	<.0001				0.28 (0.14 to 0.550)	.0002
Anxiety (HADS-A, z-score, per 1 SD)	418	0.34 (0.27 to 0.43)	<.0001				0.4 (0.26 to 0.64)	.0001
Depression (HADS-D, z-score, per 1 SD)	420	0.16 (0.11 to 0.23)	<.0001				Not selected	

Footnotes: N=117/427 survivors (27.4%) showed impaired FACT-G QOL scores. †Any relationship was defined as: relationship or married (regardless of living together) vs divorced, widowed or being single. The latter was the reference category. Abbreviations: SD – standard deviation, IC – Intensive chemotherapy, alloHSCT – allogeneic hematological stem cell transplantation, OR – odds ratio, CI – confidence interval. (i) not selected due to selection of household income

3.6. Supplementary Table S6. Univariate and multivariable logistic regression results for impaired gLS

Factor	Univariate			Entered into model building	Final multivariable model after backward selection		Final model with depression, anxiety and fatigue variables available for selection	
	N	OR (95%CI)	p		OR (95%CI)	p	OR (95%CI)	p
Sex (female vs male)	414	0.61 (0.35 to 1.07)	.08	Y	Not selected		Not selected	
Age at study participation, per 1 year	414	0.96 (0.94 to 0.98)	.0007	Y	0.96 (0.92 to 1.00)	.02	Not selected	
Age at diagnosis, per 1 year	414	0.97 (0.95 to 0.99)	.0018	N(i)				
In a relationship (yes vs no)[†]	414	0.32 (0.17 to 0.59)	.0002	Y	Not selected		Not selected	
Children (yes vs no)	414	0.34 (0.19 to 0.60)	.0003	Y	0.35 (0.15 to 0.86)	.02	0.25 (0.10 to 0.60)	.0018
Number of children, per 1	412	0.79 (0.61 to 1.02)	.06	N(ii)				
Currently smoking (yes vs no)	414	0.71 (0.29 to 1.73)	.45	N				
Education (University entrance qualification or higher vs other)	412	0.87 (0.46 to 1.64)	.66	N				
Weekly work hours, per 1 hour	388	0.99 (0.98 to 1.01)	.38	N				
Occupational situation (changed vs no change)	405	2.06 (1.15 to 3.69)	<.0001	Y	Not selected		Not selected	
Employment status (yes vs no)	411	0.58 (0.32 to 1.04)	.07	Y	Not selected		Not selected	
Household size, per 1 person	414	0.59 (0.40 to 0.88)	.0099	Y	Not selected		Not selected	
Household income, per 250 EUR	397	0.89 (0.84 to 0.95)	.0002	Y	0.90 (0.83 to 0.98)	.0126	Not selected	
Per capita income, per 250 EUR	397	0.88 (0.79 to 0.99)	.02	N(iii)				
Financial situation (worsened vs. no change or improved)	410	4.45 (2.48 to 7.97)	<.0001	Y	3.68 (1.66 to 8.13)	.0013	Not selected	
Disability (yes vs no)	396	1.30 (0.70 to 2.43)	.41	N				
Disability degree, per 10 points	389	1.08 (0.99 to 1.17)	.06	Y	Not selected		Not selected	
Relapse (yes vs no)	414	0.59 (0.28 to 1.25)	.17	Y	Not selected		Not selected	
Treatment (IC+alloHSCT vs IC)	414	1.10 (0.61 to 1.97)	.75	N				
AML type (*de novo* vs s- or t-AML)	414	0.87 (0.42 to 1.83)	.72	N				

Factor	Univariate			Entered into model building	Final multivariable model after backward selection		Final model with depression, anxiety and fatigue variables available for selection	
	N	OR (95%CI)	p		OR (95%CI)	p	OR (95%CI)	p
Time since diagnosis, per 1 year	414	0.97 (0.90 to 1.04)	.42	N				
Time since last therapy, per 1 year	283	0.99 (0.91 to 1.07)	.71	N				
Social support (z-score, per 1 SD)	410	0.38 (0.28 to 0.52)	<.0001	Y	0.46 (0.32 to 0.67)	<.0001	Not selected	
Modified Functional Comorbidity Index, per point	366	1.13 (0.97 to 1.32)	.11	Y	Not selected		Not selected	
Additional psychometric variables (Fatigue, anxiety and depression)								
General fatigue (MFI-general, z-score, per 1 SD)	407	0.41 (0.31 to 0.54)	<.0001				Not selected	
Reduced activities (MFI-activities, z-score, per 1 SD)	407	0.44 (0.34 to 0.58)	<.0001				Not selected	
Physical fatigue (MFI-physical, z-score, per 1 SD)	408	0.40 (0.30 to 0.52)	<.0001				0.56 (0.37 to 0.85)	.0067
Mental fatigue (MFI-mental, z-score, per 1 SD)	411	0.60 (0.48 to 0.74)	<.0001				Not selected	
Reduced motivation (MFI-motivation, z-score, per 1 SD)	408	0.32 (0.23 to 0.44)	<.0001				Not selected	
Anxiety (HADS-A, z-score, per 1 SD)	405	0.54 (0.44 to 0.68)	<.0001				Not selected	
Depression (HADS-D, z-score, per 1 SD)	407	0.29 (0.21 to 0.40)	<.0001				0.36 (0.23 to 0.55)	<.0001

Footnotes: N= 57/414 (13.8%) showed impaired gLS scores. [†]Any relationship was defined as: relationship or married (regardless of living together) vs divorced, widowed or being single. The latter was the reference category. Abbreviations: SD – standard deviation, IC – Intensive chemotherapy, alloHSCT – allogeneic hematological stem cell transplantation, OR – odds ratio, CI – confidence interval. Candidate variables for multivariable model (i) not selected due to strong correlation with age. (ii) not selected due to selection of binary variable children (iii) not selected due to selection of household income.

3.7. Supplementary Table S7. Univariate and multivariable logistic regression results for impaired hrLS

Factor	Univariate			Entered into model building	Final multivariable model after backward selection		Final model with depression, anxiety and fatigue variables available for selection	
	N	OR (95%CI)	p		OR (95%CI)	p	OR (95%CI)	p
Sex (female vs male)	422	1.63 (0.96 to 2.76)	.07	Y	2.16 (1.05 to 4.45)	.04	2.93 (1.21 to 7.08)	.02
Age at study participation, per 1 year	422	1.00 (0.98 to 1.02)	.67	N				
Age at diagnosis, per 1 year	422	1.00 (0.98 to 1.01)	.63	N				
In a relationship (yes vs no)[†]	422	0.57 (0.32 to 1.03)	.06	Y	not selected		not selected	
Children (yes vs no)	422	0.71 (0.40 to 1.24)	.23	N				
Number of children, per 1	420	0.86 (0.69 to 1.07)	.18	Y	0.69 (0.48 to 0.97)	.03	not selected	
Currently smoking (yes vs no)	422	1.27 (0.64 to 2.54)	.50	N				
Education (University entrance qualification or higher vs other)	419	1.53 (0.80 to 2.93)	.19	Y	not selected		not selected	
Weekly work hours, per 1 hour	395	0.97 (0.96 to 0.99)	.0033	N(i)				
Occupational situation (changed vs no change)	410	2.57 (1.52 to 4.37)	.0005	Y	not selected		not selected	
Employment status (yes vs no)	417	0.58 (0.35 to 0.99)	.04	Y	not selected		not selected	
Household size, per 1 person	422	0.82 (0.60 to 1.12)	.21	N				
Household income, per 250 EUR	402	0.91 (0.87 to 0.96)	.0006	Y	not selected		not selected	
Per capita income, per 250 EUR	402	0.83 (0.74 to 0.93)	.0012	N(ii)				
Financial situation (worsened vs. no change or improved)	417	3.34 (1.98 to 5.61)	<.0001	Y	3.30 (1.62 to 6.69)	.0010	not selected	
Disability (yes vs no)	403	2.62 (1.38 to 4.99)	.0033	N				
Disability degree, per 10 points	395	1.14 (1.06 to 1.23)	.0004	Y	1.15 (1.04 to 1.28)	.0088	not selected	
Relapse (yes vs no)	422	0.66 (0.35 to 1.26)	.20	N				
Treatment (IC+alloHSCT vs IC)	422	0.49 (0.30 to 0.82)	.0061	Y	0.19 (0.09 to 0.41)	<.0001	0.20 (0.08 to 0.51)	.0007
AML type (*de novo* vs s- or t-AML)	422	1.03 (0.52 to 2.04)	.93	N				

Factor	Univariate			Entered into model building	Final multivariable model after backward selection		Final model with depression, anxiety and fatigue variables available for selection	
	**N**	**OR (95%CI)**	**p**		**OR (95%CI)**	**p**	**OR (95%CI)**	**p**
Time since diagnosis, per 1 year	422	1.01 (0.94 to 1.07)	.85	N				
Time since last therapy, per 1 year	287	0.96 (0.88 to 1.04)	.31	N				
Social support (z-score, per 1 SD)	418	0.51 (0.39 to 0.66)	<.0001	Y	0.53 (0.37 to 0.74)	.0003	not selected	
Modified Functional Comorbidity Index, per point	374	1.40 (1.22 to 1.60)	<.0001	Y	1.32 (1.10 to 1.58)	.0028	1.40 (1.12 to 1.74)	.0026
Additional psychometric variables								
General fatigue (MFI-general, z-score, per 1 SD)	416	0.37 (0.28 to 0.48)	<.0001				not selected	
Reduced activities (MFI-activities, z-score, per 1 SD)	415	0.35 (0.27 to 0.46)	<.0001				not selected	
Physical fatigue (MFI-physical, z-score, per 1 SD)	416	0.29 (0.22 to 0.39)	<.0001				0.40 (0.26 to 0.62)	<.0001
Mental fatigue (MFI-mental, z-score, per 1 SD)	420	0.50 (0.40 to 0.62)	<.0001				not selected	
Reduced motivation (MFI-motivation, z-score, per 1 SD)	417	0.27 (0.19 to 0.37)	<.0001				0.45 (0.27 to 0.75)	.0023
Anxiety (HADS-A, z-score, per 1 SD)	415	0.42 (0.34 to 0.53)	<.0001				0.51 (0.36 to 0.73)	.0002
Depression (HADS-D, z-score, per 1 SD)	416	0.28 (0.21 to 0.38)	<.0001				not selected	

Footnotes: N=74/422 (17.5%) showed impaired hrLS scores. [†]Any relationship was defined as: relationship or married (regardless of living together) vs divorced, widowed or being single. The latter was the reference category. Abbreviations: SD – standard deviation, IC – Intensive chemotherapy, alloHSCT – allogeneic hematological stem cell transplantation, OR – odds ratio, CI – confidence interval.
candidate variable for multivariable model; (i) not selected due to strong correlation with age; (ii) not selected due to selection of household income.

3.8. Supplementary Table S8. Crude analysis of the association between allogenic HSCT and prior AML relapse and impaired quality of life or life satisfaction

n/N(%)	Impaired gLS (57/414)	Impaired hrLS (74/422)	Impaired QoL (117/427)
**Relapse**			
no	48/319 (15.1%)	61 / 324 (18.8%)	95/328 (29.0%)
yes	9/95 (9.5%)	13 / 98 (13.3%)	22/99 (22.2%)
	**p = .16**	**p = .20**	**p = .19**
**Treatment**			
Chemotherapy + allo HSCT	37/261 (14.2%)	36/265 (13.6%)	74 / 269 (27.5%)
Chemotherapy alone	20/153 (13.1%)	38/157 (24.2%)	43/158 (27.2%)
	**p = .75**	**p = .0056**	**p = .95**

Footnotes: p-values: Chi-Squared tests.

We found that a relapse after initial diagnosis was not associated with impaired general or health related LS within the survivor cohort. With respect to treatment, survivors who underwent an allogeneic HSCT showed less often impaired hrLS compared to survivors who were treated with chemotherapy alone (p=.0056).

4. Supplementary Figures

4.1. Supplementary Figure S1. Graph of the predefined sequential testing procedure

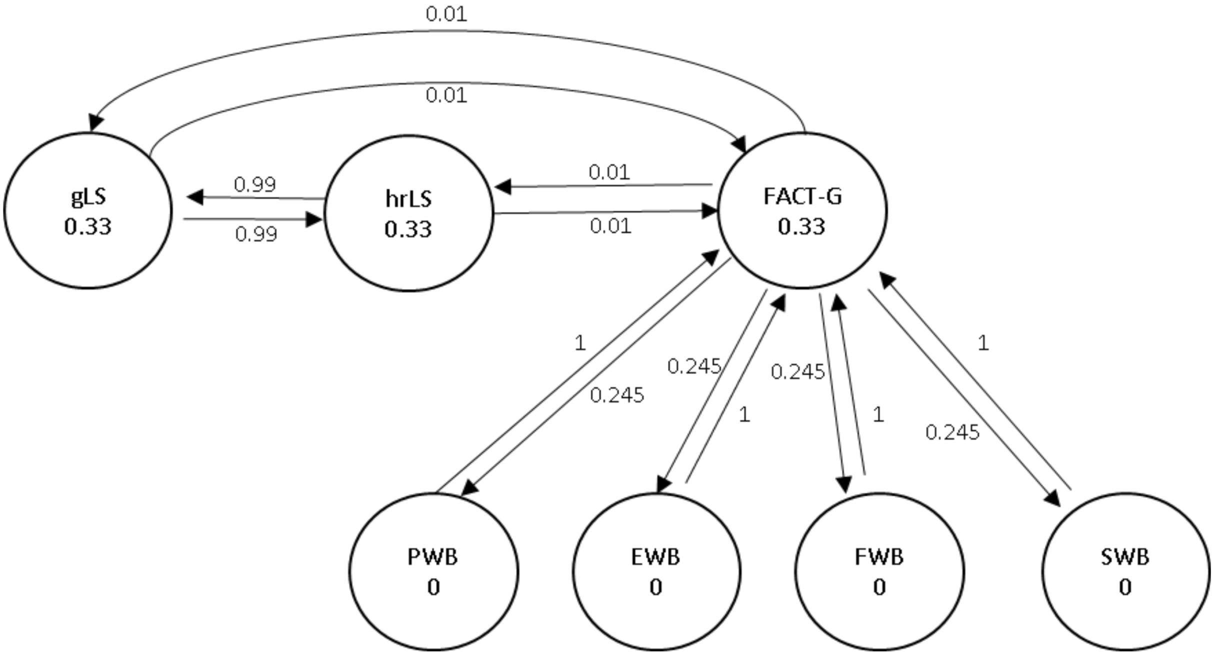

The initial predefined sequential graphical testing procedure (SRTP). The graph-based procedure consists of elementary null-hypotheses displayed as circles and dependencies between null-hypotheses as edges. Each null-hypothesis was assigned an initial weight (displayed with the circle) and each edge was assigned an initial weight displayed at the respective edge. In case of a rejected null-hypothesis the local significance level (significance level times weight) can be distributed among all connected null-hypotheses via the edges. Details on the general mechanism of the update algorithm can be found in the seminal publications by Bretz et al.

The rationale for this setup of the SRTP was based on an initial test of the z-scores of FACT-G, hrLS and gLS, as a first layer, before FACT-G subscores will be tested. hrLS and gLS were basically implemented as in a Bonferroni-Holm setting. hrLS, gLS and FACT-G at start of the procedure are assigned 1/3 of the global significance level of 0.05. FACT-G subscores did not get any weight (w=0). After FACT-G gets rejected weights will be propagated among the predefined edges and edge weights if a null hypothesis can be rejected, then the remaining significance level will be distributed equally among the four subscores PWB (physical well-being), EWB (emotional well-being), FWB (functional well-being) and SWB (social well-being).

4.2. Supplementary Figure S2. Distribution of QoL (FACT-G) and life satisfaction (gLS, hLS) by age group, AML type, relapse, sex and therapy

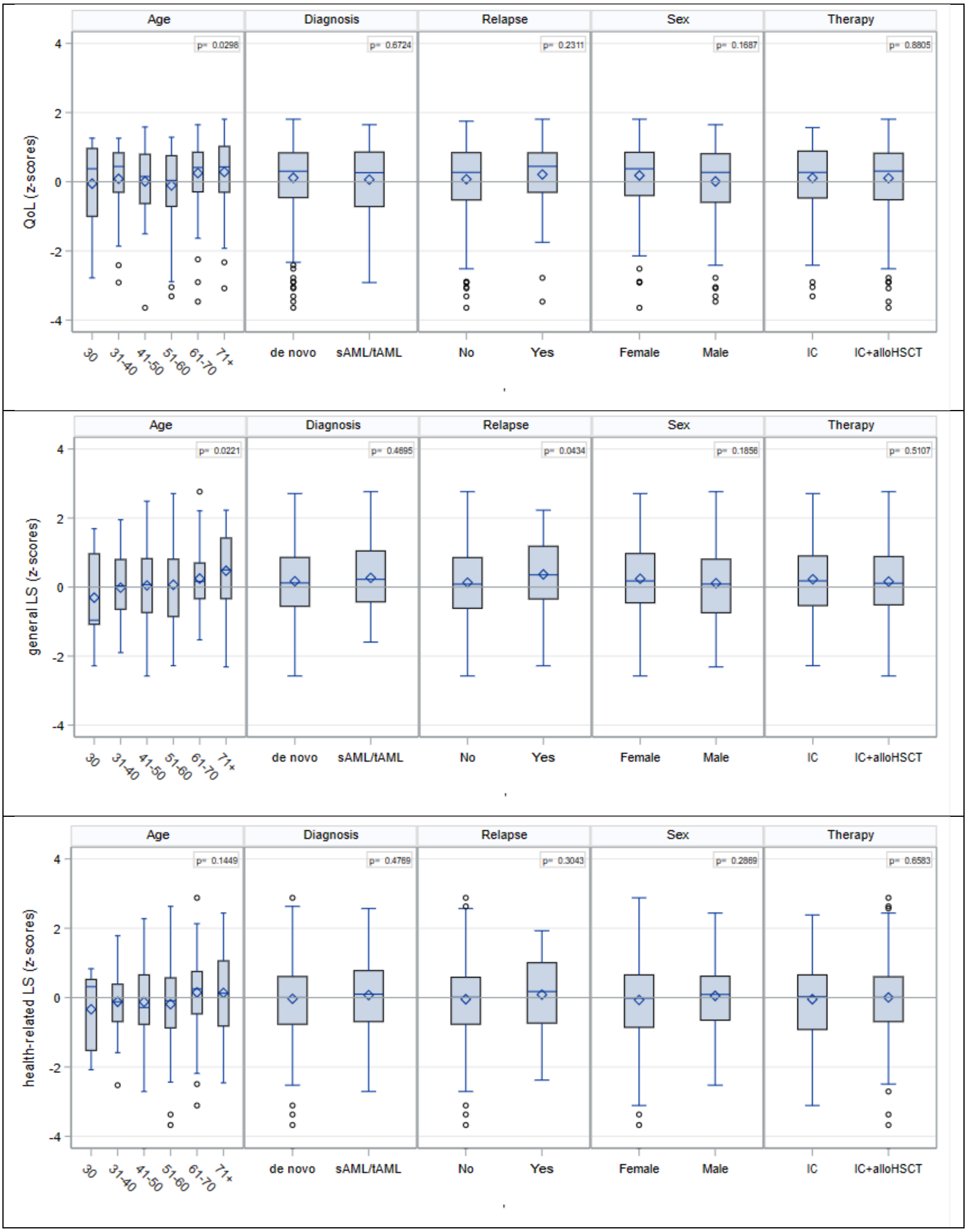

4.3. Supplementary Figure S3: Density plots of outcomes on FACT-G subscales

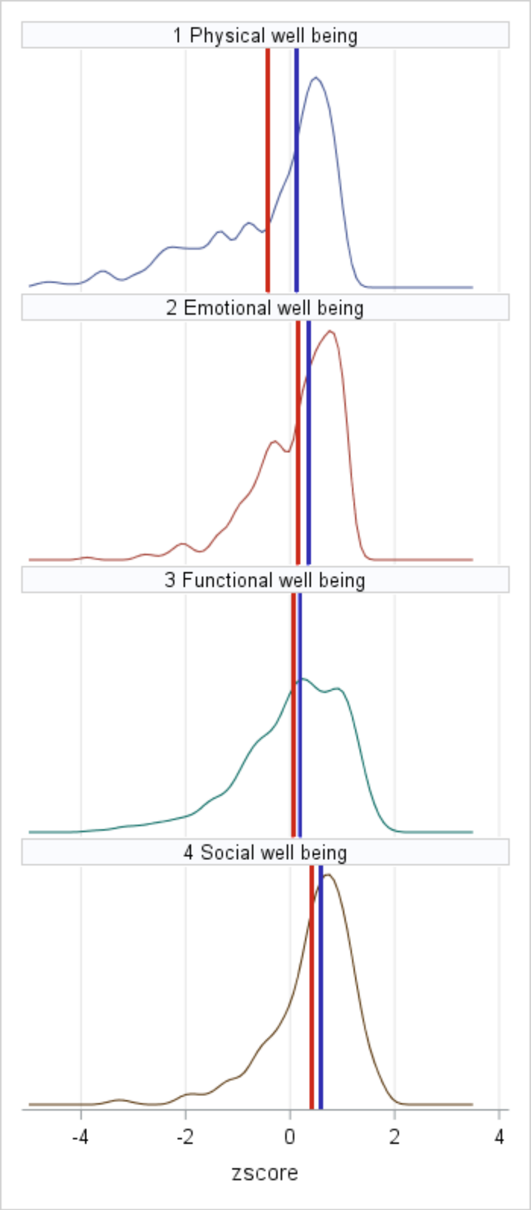

Footnotes: Red line: mean; blue line: median.

4.4. Supplementary Figure S4: Density plots of secondary outcomes

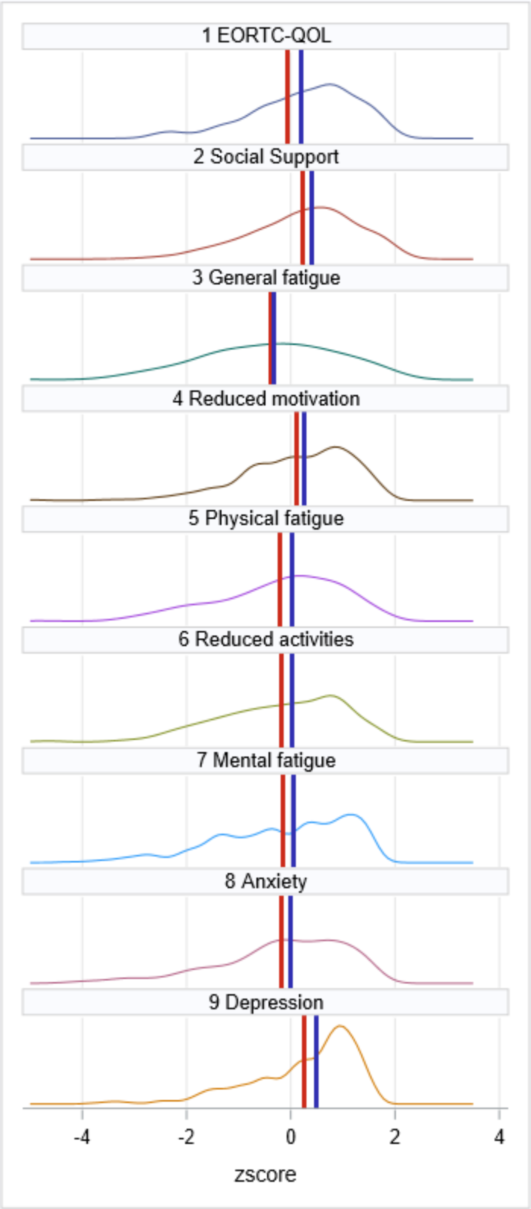

Footnotes: Red line: mean; blue line: median

5. Supplementary References

1. Bretz F, Maurer W, Brannath W, Posch M. A graphical approach to sequentially rejective multiple test procedures. Stat Med. 2009;28(4):586-604.
2. Bretz F, Posch M, Glimm E, Klinglmueller F, Maurer W, Rohmeyer K. Graphical approaches for multiple comparison procedures using weighted Bonferroni, Simes, or parametric tests. Biom J. 2011;53(6):894-913.
3. Holzner B, Kemmler G, Cella D, De Paoli C, Meraner V, Kopp M, et al. Normative data for functional assessment of cancer therapy--general scale and its use for the interpretation of quality of life scores in cancer survivors. Acta Oncol. 2004;43(2):153-60.
4. Yost KJ, Eton DT. Combining Distribution- and Anchor-Based Approaches to Determine Minimally Important Differences:The FACIT Experience. Evaluation & the Health Professions. 2005;28(2):172-91.
5. Groll DL, To T, Bombardier C, Wright JG. The development of a comorbidity index with physical function as the outcome. J Clin Epidemiol. 2005;58(6):595-602.
6. Cella DF, Tulsky DS, Gray G, Sarafian B, Linn E, Bonomi A, et al. The Functional Assessment of Cancer Therapy scale: development and validation of the general measure. J Clin Oncol. 1993;11(3):570-9.
7. Henrich G, Herschbach P. Questions on Life Satisfaction (FLZM) - A Short Questionnaire for Assessing Subjective Quality of Life. European Journal of Psychological Assessment. 2000;16(3):150-9.
8. Daig I, Spangenberg L, Henrich G, Herschbach P, Kienast T, Brähler E. Alters- und geschlechtspezifische Neunormierung der Fragen zur Lebenszufriedenheit (FLZM) für die Altersspanne von 14 bis 64 Jahre. [Age and gender specific new normative data for the Questions on Life Satisfaction Questionnaire for 14–64 years olds.]. Zeitschrift für Klinische Psychologie und Psychotherapie: Forschung und Praxis. 2011;40(3):172-8.
9. Fayers P, Bottomley A. Quality of life research within the EORTC-the EORTC QLQ-C30. European Organisation for Research and Treatment of Cancer. Eur J Cancer. 2002;38 Suppl 4:S125-33.
10. Nolte S, Waldmann A, Liegl G, Petersen MA, Groenvold M, Rose M. Updated EORTC QLQ-C30 general population norm data for Germany. Eur J Cancer. 2020;137:161-70.
11. Zigmond AS, Snaith RP. The hospital anxiety and depression scale. Acta Psychiatr Scand. 1983;67(6):361-70.
12. Herrmann-Lingen C, Buss U, Snaith P, Herrmann-Lingen C. Hospital anxiety and depression scale deutsche Version; HADS-D. 3., aktual. u. neu normierte Aufl. ed. Huber Bern: Huber; 2011.
13. Hinz A, Brähler E. Normative values for the hospital anxiety and depression scale (HADS) in the general German population. J Psychosom Res. 2011;71(2):74-8.
14. Smets EM, Garssen B, Bonke B, De Haes JC. The Multidimensional Fatigue Inventory (MFI) psychometric qualities of an instrument to assess fatigue. J Psychosom Res. 1995;39(3):315-25.
15. Schwarz R, Krauss O, Hinz A. Fatigue in the general population. Onkologie. 2003;26(2):140-4.
16. Dalgard OS, Bjørk S, Tambs K. Social support, negative life events and mental health. Br J Psychiatry. 1995;166(1):29-34.
17. Kocalevent RD, Berg L, Beutel ME, Hinz A, Zenger M, Härter M, et al. Social support in the general population: standardization of the Oslo social support scale (OSSS-3). BMC Psychol. 2018;6(1):31.
18. (Hrsg) RK-I. Daten und Fakten: Ergebnisse der Studie »Gesundheit in Deutschland aktuell 2012«. Beiträge zur Gesundheitsberichterstattung des Bundes, RKI, Berlin 2014.
